# Masculinity Barriers to Ever Completing Colorectal Cancer Screening among American Indian/Alaska Native, Black, and White Men (Ages 45–75)

**DOI:** 10.3390/ijerph19053071

**Published:** 2022-03-05

**Authors:** Charles R. Rogers, David G. Perdue, Kenneth Boucher, Kevin M. Korous, Ellen Brooks, Ethan Petersen, John M. Inadomi, Fa Tuuhetaufa, Ronald F. Levant, Electra D. Paskett

**Affiliations:** 1 Department of Family & Preventive Medicine, University of Utah School of Medicine, Salt Lake City, UT 84108, USA; kevin.korous@utah.edu (K.M.K.); ellen.brooks@utah.edu (E.B.); ethan.petersen@hsc.utah.edu (E.P.); u0796061@utah.edu (F.T.); 2 MNGI Digestive Health, Minneapolis, MN 55413, USA; david.perdue@mngi.com; 3 Cancer Biostatistics Shared Resource, Huntsman Cancer Institute, Salt Lake City, UT 84112, USA; u0030602@umail.utah.edu; 4 Department of Internal Medicine, University of Utah School of Medicine, Salt Lake City, UT 84132, USA; john.inadomi@hsc.utah.edu; 5 Department of Psychology, The University of Akron, Akron, OH 44325, USA; levant@uakron.edu; 6 Department of Internal Medicine, College of Medicine, The Ohio State University, Columbus, OH 43210, USA; electra.paskett@osumc.edu

**Keywords:** colonic neoplasms, men’s health, early detection of cancer, minority health, Indigenous peoples, health equity

## Abstract

Disparities in colorectal cancer (CRC) mortality among White, Black, and American Indian/Alaska Native (AIAN) men are attributable to differences in early detection screening. Determining how masculinity barriers influence CRC screening completion is critical for cancer prevention and control. To determine whether masculinity barriers to medical care are associated with lower rates of ever completing CRC screening, a survey-based study was employed from December 2020–January 2021 among 435 White, Black, and AIAN men (aged 45–75) who resided in the US. Logistic regression models were fit to four Masculinity Barriers to Medical Care subscales predicting ever completing CRC screening. For all men, *being strong* was associated with 54% decreased odds of CRC screening completion (OR 0.46, 95% CI 0.23 to 0.94); each unit increase in *negative attitudes toward medical professionals and exams* decreased the odds of ever completing CRC screening by 57% (OR 0.43, 95% CI 0.21 to 0.86). Black men who scored higher on *negativity toward medical professionals and exams* had decreased odds of ever screening. Consideration of masculinity in future population-based and intervention research is critical for increasing men’s participation in CRC screening, with more salience for Black men.

## 1. Introduction

Since the mid-1990s, colorectal cancer (CRC) incidence and mortality have declined significantly in adults aged 50 years and older [1,2,3], but increased among those under age 50 (early-onset CRC (EOCRC)). By 2030, EOCRC incidence is predicted to increase by 90% [4]. Of an estimated 147,950 new CRC cases diagnosed in 2020, 12% occurred among individuals aged under 50 years [1,2]. 

CRC mortality is largely preventable with regular screening, yet American Indian/Alaska Native (AIAN) and Black men [2,4,5,6,7,8,9] in the US continue to experience higher CRC incidence and mortality than their White male counterparts [1,5,7,10]. Studies have found 24% higher CRC incidence [10] and 36% to 47% higher mortality in Black men than in White men [2,10]. For AIAN men, incidence and mortality are approximately 4% and 3% higher than for White men, but regional variation is substantial, being lower in the east and southwest (30 to 40 per 100,000) to more than double for those residing in Alaska (95 per 100,000) [1,2]. Black and AIAN men are also more likely to be diagnosed with later-stage CRC [11]. Racial misclassification in cancer registries and death records likely underestimates the true extent of cancer disparities in these populations [12,13]. 

Disparities also exist for EOCRC incidence, which between 1973 and 1994 was 53.4% higher in Blacks than in Whites [14]. Due to rapid increases in EOCRC incidence among White (2% annually) and AIAN people (2.2% annually) between 2012 and 2016, EOCRC incidence is now comparable between Blacks and Whites (12.7 vs. 11.0 per 100,000), followed closely by AIAN people; however, disparities still exist [1,15,16]. Rising EOCRC incidence led to calls to reduce the recommended age of CRC screening initiation from 50 to 45 years for average-risk adults, regardless of race [17,18,19]. The American Cancer Society endorsed this change in 2018 [20], and the US Preventive Services Task Force followed suit in 2021 [21].

Significant racial disparities in CRC screening are well documented [2,6,8,10,22]. An analysis of cancer screening test use among individuals aged 50 to 75 years found that 63.7% of White men adhered to CRC screening guidelines compared with 59.3% of Black men and 48.4% of AIAN men [22]. Lack of access to quality care and racial bias in treatment also contribute to low rates of CRC screening among Black men through perpetuation of medical mistrust and a decreased likelihood of utilizing medical services [23,24,25,26,27]. Similarly, AIAN men often must navigate healthcare discrimination, cultural insensitivity, language barriers, poor access to care, and poorly funded Indian Health Service (IHS), tribal, and urban health systems—all of which can contribute to lower screening completion rates [2,28,29,30,31].

It is also important to consider how psychosocial determinants of men’s health may influence their screening participation rates [29,30,32,33,34,35,36,37]. Endorsement of traditional masculinity and gender norms is associated with both decreased participation in preventive health behaviors and negative attitudes toward asking for help and behaviors perceived as vulnerable [38,39,40]. To support cancer prevention and control efforts, elucidation of how masculinity barriers to medical care intersect with psychosocial determinants of men’s health to exacerbate inequalities in CRC incidence and mortality is warranted.

The COVID-19 pandemic has exacerbated cancer health disparities, underscored existing health injustices among Black and AIAN people, and widened the life-expectancy gap among Black, AIAN, and White populations [2,41,42,43]. Lockdowns and forced closures of clinics and community health centers have worsened vulnerable communities’ already limited access to preventive cancer screenings and other health services [42,44]. AIAN communities have been particularly affected by limited IHS resources to care for individuals with COVID-19 [45,46].

Delayed CRC screening results in later-stage disease diagnosis and increased mortality [44]. Research is necessary to elucidate the factors leading to disparate CRC screening rates and inequitable healthcare access and utilization. We undertook this study to determine whether masculinity barriers to medical care influence early-detection CRC screening completion among Black and AIAN men aged 45 to 75 years compared with White men. Our hypothesis was that a higher endorsement of masculinity barriers to medical care would be associated with reduced CRC screening uptake.

## 2. Materials and Methods

### 2.1. The Study Design and Setting

We used a survey-based, cross-sectional design to analyze the association between masculinity barriers and ever completing a CRC screening test. In partnership with Qualtrics (Provo, UT), a commercial survey sampling and administration company, participants were recruited between December 2020 and January 2021 using a nationwide consumer-panel sampling approach. Recruitment sources included targeted email lists, member referrals, permission-based networks, customer loyalty web portals, and social media. This study was approved by the Institutional Review Board (IRB) at the University of Utah (IRB #00113679) before data collection. All participants provided informed consent, were invited to choose a method of compensation (e.g., frequent flier miles, points toward retail purchases), and then directed to the survey. The 73-question online survey took up to 15 min to complete via a smart phone or tablet.

### 2.2. Participants

Male respondents aged 18 to 75 years from across the US who self-identified as Black, AIAN, or White were eligible to complete an online survey designed to investigate multiple hypotheses (e.g., CRC screening intention versus completion). For this study, given our hypothesis on CRC screening completion, we included only participants who self-identified as aged 45 years or older. Of 870 men who consented to the survey, 191 (22%) were excluded after failing the eligibility check; of the remaining 679 men, 42 (6%) were excluded after failing quality checks. Of the remaining 637 men, 435 (68%) were aged 45 to 75 years and were included in the current study.

### 2.3. Variables

The primary outcome, reporting ever completing a CRC screening test, was defined as 0 (no) or 1 (yes) in response to one of two questions: (a) “A blood stool test is a test that may use a special kit at home to determine whether the stool contains blood. Have you ever had this test using a home kit?” (b) “Sigmoidoscopy and colonoscopy are exams in which a tube is inserted in the rectum to view the colon for signs of cancer or other health problems. Have you ever had either of these exams?”. Responses of *unsure* were coded as missing. 

Masculinity barriers to CRC screening completion were treated as predictors and were measured by the reduced yet validated Masculinity Barriers to Medical Care scale, which consists of 18 items rated on a five-point Likert-type scale and has demonstrated good reliability [47]. The current study focused on the four Masculinity Barriers to Medical Care subscales: *being strong* (six items); *acknowledging emotions and health issues* (four items), *negative attitudes toward medical professionals and exams* (four items), and *positive attitudes toward medical professionals and exams* (four items). All items within each subscale were positively correlated and averaged to form a mean score for each subscale. Higher scores for *being strong* and *negative attitudes toward medical professional and exams* and lower scores for *acknowledging emotions and health issues* and *positive attitudes toward medical professionals and exams* suggest greater masculinity barriers to medical care. 

Age was dichotomized into two groups: 45 to 59 years (reference category) and 60 to 75 years. Race was categorized as Black (reference group), AIAN, or White. Five confounders were included as categorical variables (categorized to maximize sample size in each group) on the basis of their relationship to CRC screening in prior research: marital status (single, divorced, separated, or widowed vs. married or in a relationship); educational attainment (some schooling, high school diploma, or GED vs. some college or Associate’s degree, and vs bachelor’s degree or higher); health insurance status (has health insurance vs. not), regular provider (has one vs. not), and family history of CRC (has a family history vs. none or unsure). 

### 2.4. Statistical Analysis

Four participants were missing data on the primary outcome and Masculinity Barriers to Medical Care subscales and were excluded from the analysis using a listwise deletion approach. 

All analyses were performed in R version 4.0.2. Descriptive data were examined across participant demographics, using frequencies. For each Masculinity Barriers to Medical Care subscale, the mean, standard deviation, and Cronbach’s alpha were computed, as well as between-subscale correlations. A series of logistic regression models were specified separately for each of the four Masculinity Barriers to Medical Care subscales predicting ever completing CRC screening. To control for confounding effects, we included all five adjustment variables in each model. Age and race were included in all models. Initially, we included an interaction term between age and each Masculinity Barriers to Medical Care subscale; finding no evidence of an interaction, we excluded the age interaction term from the final models to reduce the number of predictors. The interaction term between race and the Masculinity Barriers to Medical Care subscale was included in all models to test for effect modification. A two-sided *p*-value < 0.05 was considered significant. Standardized betas were used to calculate odds ratios (ORs) and their 95% confidence intervals (CIs). CIs that did not overlap with zero provided evidence of an effect. In the presence of effect modification, we used relative excess risk due to interaction (RERI) as a measure of the additive scale [48].

### 2.5. Sample Size Considerations

We included all participants who met the eligibility criteria. A priori power analyses were not conducted; however, we conducted a sensitivity power analysis using G*Power version 3.1.9.4 [49]. On the basis of our sample size after examining missingness (431), we had 80% power to detect ORs ≤0.75 and ≥1.34, if participants with mean scores on the Masculinity Barriers to Medical Care subscales had a 35% probability of ever completing CRC screening. At a higher CRC screening completion rate for mean scores (50% probability), we had 80% power to detect ORs ≤0.76 and ≥1.32.

## 3. Results

### 3.1. Sample Characteristics

Table 1 summarizes the demographic characteristics of the 435 participants who met all inclusion criteria. The sample was nearly evenly distributed between the two age groups. More White men completed the survey (45%) than AIAN (31%) or Black men (23%). Across participants, 68% reported having ever completed CRC screening. Most of the sample was married or in a relationship, had at least some college education, and had health insurance, a regular provider, and no family history of CRC.

Table 2 summarizes scores for each Masculinity Barriers to Medical Care subscale. The mean score across subscales ranged from 2.05 to 3.61; measurement reliability ranged from 0.53 to 0.67. Subscales were positively correlated (range of *r* = 0.06 to 0.29) apart from the correlation between *negative* and *positive attitudes toward medical professionals and exams* (*r* = −0.16). 

### 3.2. Main Analyses

Table 3 presents ORs for each of the four models. There was no evidence of an association between responses to *acknowledging emotions and health issues* or *positive attitudes toward medical professionals and exams* and ever completing CRC screening. However, endorsing questions about *being strong* and *negative attitudes toward medical professionals and exams* was associated with lower reported CRC screening. A unit increase on the *being strong* subscale was associated with a 54% decrease in the odds of ever completing a test, while a unit increase on the *negative attitudes toward medical professionals and exams* subscale was associated with a 57% decrease in the odds of doing so. Participants aged 60 to 75 years had consistently higher odds of ever completing CRC screening. Neither model showed race being independently associated with ever completing CRC screening. However, there was evidence of effect modification by race for *negative attitudes toward medical professionals and exams* (Table 4). Black men with higher scores on this subscale had decreased odds of ever completing CRC screening. However, we found no evidence of a difference in ever completing CRC screening among AIAN men when we stratified by this subscale. Among the examined confounders, being married or in a relationship and having a regular provider were associated with higher odds of ever completing CRC screening. 

### 3.3. Sensitivity Analyses

As a sensitivity test, we reran the model containing a subset of the adjustment variables selected using lasso regression (e.g., marital status, insurance status, regular provider). Results for the smaller adjusted model were like those for the fully adjusted model, with one exception (see Appendix A Table A1 and Table A2). Compared with Black men, AIAN men had 89% lower odds of ever completing CRC screening; however, because the CI for this OR ranged from 0.01 to 0.94 in this adjusted model and from 0.01 to 1.02 in the fully adjusted model, the magnitude of the difference is unclear.

## 4. Discussion

In this population-based survey study, we aimed to determine whether masculinity barriers to medical care influenced ever completing CRC early-detection screening among Black and AIAN men aged 45 to 75 years compared with their White counterparts. Results indicated that men in the older age group (60–75 years) were more likely to report ever completing CRC screening. When considering masculinity barriers, the models *being strong* and *negative attitudes* were associated with decreased odds of ever completing CRC screening among all men. Interestingly, the decreased odds associated with *negative attitudes* were more prevalent among Black men but not among AIAN men.

In all models, older age (60–75 years) was associated with higher odds of ever completing CRC screening compared with younger age (45–59 years). CRC risk increases with age, and other studies have confirmed the relationship between aging and CRC screening behavior [50,51,52]. Furthermore, these men have been screening eligible for longer periods of time. However, the younger age cohort included men 45–49 years old, which may have contributed to the age difference in CRC completion. Future research should focus on increasing screening uptake among younger men to address their increasing CRC rates [15,53]. 

Modeling estimates predict approximately 4500 excess CRC deaths between 2020 and 2030 due to COVID-19-driven delays in cancer-related services [44,54,55]. It is unknown the impact of the concurrent reduction in the recommended age of CRC screening initiation to 45 years, but COVID-delayed screening may have disproportionately reduced the odds of ever completing CRC screening among younger individuals in our sample who had less time to be screening-eligible and we asked about ever having been screened. Moreover, younger Black patients with CRC have been shown to have worse treatment outcomes at every disease stage [56]; hence, other social determinants of health may also play a role and must be considered in future disparities-focused research.

Consistent with prior literature, this study observed that masculinity barriers interfered with ever completing CRC screening. Specifically, the constructs *being strong* and *negative attitudes* were associated with lower odds of ever completing CRC screening. Men who strongly endorse masculine ideals have been reluctant to seek and engage in preventive health services, including CRC screening, for fear of displaying weakness and vulnerability [33,40,57,58]. Additionally, masculine ideologies positively correlate with medical mistrust [59], underscoring that hesitancy in seeking medical care can negatively affect provider-patient trust building through decreased interaction [60,61]. 

Our study also revealed that Black men scoring higher on the *negative attitudes toward medical professionals and exams* subscale had lower odds of CRC screening uptake, while no association was seen for the subscales *positive attitudes* and *acknowledging emotions and health issues*. A previous systematic review of CRC screening barriers found that Black men harbored greater mistrust of healthcare systems and providers than Black women and highlighted the need for qualitative data exploring the impact of gender norms, including masculinity, on this relationship [34]. This suggests that, while mistrust remains a significant barrier to CRC screening for Black men, having trust does not overcome other barriers. Warranting further investigation, medical mistrust related to COVID-19 may have deterred Black men from being screened for CRC, as has been seen with COVID-19 treatment and vaccine hesitancy among Black people nationally [59,62,63]. 

An avenue for further investigation entails determining if providers should consider recommending noninvasive stool tests for CRC as alternatives to colonoscopies among Black men, as suggested by Shaukat et al. [64]; this may reduce masculinity barriers to CRC screening and increase the odds of uptake of these noninvasive modalities as Black men feel more empowered to take ownership of their CRC-related health. It is important to consider how reframing masculinity-related messages may influence men’s CRC screening completion. Research indicates that using a gain-framed approach to health-related behavior change may be more effective than loss-framed messaging [65,66]. Among Black men in particular, gain-framed messaging has been associated with greater receptivity to CRC screening; however, this finding warrants further study [67].

Surprisingly, ever completing CRC screening did not correlate with positive or negative attitudes toward medical professionals among AIAN participants, who have for decades had among the poorest documented overall health outcomes of all racial and ethnic groups in the US [68] Yet, our sensitivity analysis suggested that AIAN men had lower odds of ever completing CRC screening compared to Black men, although this warrants further replication. This contrasts with qualitative studies that have shown medical mistrust to be a significant barrier to screening in this population [29,36]. These findings may identify an important distinction between focus groups and other qualitative study methodologies that emphasize health perceptions and screening intent versus actual completion of screening [69,70]. Alternatively, this may be a function of the AIAN cohort in this study, which—on the basis of a high prevalence of insurance, educational attainment, and having a regular health provider—may differ from the AIAN populations commonly engaged in population-based research. Because individuals without consistent healthcare or insurance often have significantly lower CRC screening rates [1,22], more community-based participatory research should emphasize increasing awareness of and access to CRC-related information and screening availability to decrease CRC mortality among tribal communities. 

### Limitations

This study had several limitations. Firstly, since most participants had health insurance and at least some college education, our sample may not be generalizable to the entire US population, and we may not have captured the influence of socioeconomic factors that can interfere with CRC screening [4]. Secondly, the reliability coefficients for the Masculinity Barriers to Medical Care subscales were acceptable but not optimal. Smaller coefficients could have resulted from estimating reliability across the entire sample, as the initial purpose of the Masculinity Barriers to Medical Care scale was to capture masculinity barriers among Black men [47]. Thirdly, our outcome was informed by self-reports of ever having completed a CRC screening test; there are potential biases inherent in self-report measures, and ever completing CRC screening does not account for being current with screening. Lastly, given our sample size, we collapsed across some categories of confounders to improve the stability of our models. 

## 5. Conclusions

This study is the first to examine the association between masculinity barriers to medical care and ever completing CRC screening among Black, AIAN, and White men, including those under age 50, and it provides a foundation for future population-based and intervention research. Since 45 years of age is now the age to begin early detection screening for CRC among all average risk adults, the conversation about this important policy change between healthcare providers and Black, AIAN, and White men must begin earlier, and masculinity barriers to medical care discussed in our study must be considered. 

## Figures and Tables

**Table 1:** Summary of demographic characteristics.

Characteristics	*N* (%)
Total Men	435 (100)
Colorectal cancer screening status (ever)	
Yes	294 (68)
No	137 (31)
Age	
45–59	215 (49)
60–75	220 (51)
Race	
Black	99 (23)
AIAN	136 (31)
White	200 (46)
Marital status	
Married or in a relationship	263 (60)
Single, separated, divorced, or widowed	170 (39)
Educational attainment	
Highschool, GED, or less	90 (21)
Some college or Associate’s degree	166 (38)
Bachelor’s degree or higher	177 (41)
Health insurance	
Yes	385 (89)
No	47 (11)
Regular provider	
Yes	363 (83)
No	69 (16)
Family history of colorectal cancer	
Yes	33 (8)
No/unsure	399 (92)

**Table 2:** Summary of the Masculinity Barriers to Medical Care subscales (*N* = 431).

Subscale				Correlations			
	*M*	*SD*	Cronbach’s Alpha	1	2	3	4
1. Being strong	3.61	0.72	0.63	-			
2. Acknowledging emotions and health issues	3.38	0.86	0.67	0.26	-		
3. Negative attitudes toward medical professionals and exams	2.05	0.78	0.54	0.14	0.05	-	
4. Positive attitudes toward medical professionals and exams	3.03	0.80	0.53	0.06	0.29	−0.16	-

**Table 3:** Odds ratios (ORs) for the odds of ever having completed colorectal cancer screening among Black, AIAN, and White men (fully adjusted model).

Subscale	Parameter	*OR*	95% CI
Being strong	Constant	4.13	0.14, 122.62
	**Unit increase in subscale**	**0.46**	**0.23, 0.94**
	White	0.13	0.01, 3.51
	AIAN	0.09	0.00, 2.79
	**Age (60–75)**	**3.60**	**2.26, 5.73**
	Subscale × White	1.49	0.63, 3.54
	Subscale × AIAN	1.89	0.79, 4.56
Acknowledging emotions and health issues	Constant	0.77	0.03, 19.42
	Unit increase in subscale	0.72	0.39, 1.34
	White	0.14	0.01, 2.16
	AIAN	0.35	0.02, 5.56
	**Age (60–75)**	**3.53**	**2.22, 5.59**
	Subscale × White	1.52	0.73, 3.15
	Subscale × AIAN	1.32	0.62, 2.79
Negative attitudes toward medical professionals and exams	Constant	2.53	0.15, 42.45
	**Unit increase in subscale**	**0.43**	**0.21, 0.86**
	White	0.16	0.02, 1.16
	AIAN	0.12	0.01, 1.02
	**Age (60–75)**	**3.08**	**1.92, 4.96**
	Subscale × White	1.72	0.76, 3.87
	**Subscale × AIAN**	**2.40**	**1.01, 5.71**
Positive attitudes toward medical professionals and exams	Constant	0.23	0.02, 3.21
	Unit increase in subscale	0.97	0.57, 1.66
	White	0.38	0.04, 3.38
	AIAN	0.36	0.04, 3.45
	**Age (60–75)**	**3.47**	**2.19, 5.50**
	Subscale × White	1.19	0.58, 2.41
	Subscale × AIAN	1.43	0.67, 3.07

Black men aged 45–59 were specified as the reference group (constant). ORs are adjusted for marital status, education, insurance status, regular provider, and family history of colorectal cancer. Bold text highlights significant predictors.

**Table 4:** Modification table of the effect of negative attitudes toward medical professionals and exams on ever completing a colorectal cancer screening test between Black and AIAN men (fully adjusted model).

	*N*, No/Yes CRCS	Not at All True	One-Unit Increase
		OR 95% CI	OR 95% CI
**Black men**	27/20	1.0	0.43 0.21, 0.86
AIAN men	40/94	0.12 0.01, 1.02	1.03 0.61, 1.75

Measure of effect modication on additive scale: RERI (95% CI)] = 0.57 (0.28, 0.87). ORs are adjusted for marital status, education, insurance status, regular provider, and family history of CRC.

## Data Availability

The data presented in this study are available on request from the corresponding author.

## Appendix A

**Table A1:** Odds ratios (ORs) for the odds of ever having completed colorectal cancer screening among Black, AIAN, and White men (smaller adjusted model).

Subscale	Parameter	*OR*	95% CI
Being strong	Constant	2.45	0.10, 61.98
	**Unit increase in subscale**	**0.47**	**0.23, 0.96**
	White	0.13	0.00, 3.46
	AIAN	0.09	0.00, 2.82
	**Age (60–75)**	**3.66**	**2.31, 5.82**
	Subscale × White	1.50	0.63, 3.56
	Subscale × AIAN	1.87	0.78, 4.52
Acknowledging emotions and health issues	Constant	0.43	0.02, 8.90
	Unit increase in subscale	0.74	0.40, 1.37
	White	0.15	0.01, 2.24
	AIAN	0.37	0.02, 5.83
	**Age (60–75)**	**3.60**	**2.27, 5.69**
	Subscale × White	1.50	0.72, 3.10
	Subscale × AIAN	1.29	0.61, 2.73
Negative attitudes toward medical professionals and exams	Constant	1.59	0.12, 21.79
	**Unit increase in subscale**	**0.43**	**0.21, 0.86**
	White	0.14	0.02, 1.05
	**AIAN**	**0.11**	**0.01, 0.94**
	**Age (60–75)**	**3.18**	**1.98, 5.10**
	Subscale × White	1.80	0.80, 4.06
	**Subscale × AIAN**	**2.46**	**1.03, 5.84**
Positive attitudes toward medical professionals and exams	Constant	0.13	0.01, 1.50
	Unit increase in subscale	0.99	0.58, 1.70
	White	0.37	0.04, 3.25
	AIAN	0.37	0.04, 3.49
	**Age (60–75)**	**3.53**	**2.23, 5.58**
	Subscale × White	1.19	0.59, 2.41
	Subscale × AIAN	1.41	0.66, 3.03

Black men aged 45–59 were specified as the reference group. ORs are adjusted for marital status, insurance status, and regular provider. Bold text highlights significant predictors.

**Table A2:** Modification table of the effect of negative attitudes toward medical professionals and exams on ever completing a colorectal cancer screening test between Black and AIAN men (smaller adjusted model).

	*N*, No/Yes CRCS	Not at All True	One-Unit Increase
		OR 95% CI	OR 95% CI
**Black men**	27/20	1.0	0.430.21, 0.86
AIAN men	40/94	0.110.01, 0.94	1.050.62, 1.78

Measure of effect medication on additive scale: RERI (95% CI) = 0.58 (0.29, 0.87). ORs are adjusted for marital status, insurance status, and regular provider.
